# Mr. Cribb's Singular Case of Delivery

**Published:** 1805-07-01

**Authors:** W. O. Cribb

**Affiliations:** Bishops Stortford, Herts


### To Dr. BRADLEY.

SIR,

HAVING lately met with a case, which, as far as my reading or practice extends, is without its parallel, I take the liberty of communicating it to you, with leave to make it public or not, as you may think it merits the attention of the profession.

On Friday, April 27, 1804, I received a note from my friend, Mr. Cooper, an intelligent practitioner in this neighbourhood, requesting my assistance "in a very serious midwifery case." I found the woman (who had been in labour from Tuesday evening) with *strong forcing* pains, returning at short intervals. Mr. Cooper informed me, that she had been delivered of a child about nine years before; but that as there was something *extremely singular* in this case at present, he wished me to make my own observations, and to form my opinion *solely*, from such circumstances as I should observe on examination; without any bias or prejudice from his relation of it.

On examining, I found a large and hard tumour pressing very low down and covered by an unusual substance, which felt soft and uneven to the touch, and the projection of the os sacrum was much more plainly perceived than in a common examination per vaginam; yet by the yielding of the bones to a moderately firm pressure, there was but little doubt of its being the head of a child, and I could also feel the sutures, though but indistinctly. The first idea that suggested itself was, that of its being an extra-uterine fœtus; but the head was propelled too low within the brim of the pelvis, and pressed down too forcibly during the pains, to admit of this idea for a moment; and as neither the *os uteri* nor the *arch of the pubis* were to be felt, it rendered the case still more obscure, at the same time that it strengthened the opinion that it could not be extra-uterine. In order therefore to make the examination more perfect, and to ascertain as accurately as possible the precise nature of the case, I attempted to pass the index of the left hand *per anum*, whilst I continued that of the right in the vagina, when, to my very great surprise, I found that my right index was already in the anus. This at once explained the reason why I felt the sacrum so plainly, and that the membrane covering the head must be the intestinum rectum and vagina; and it satisfactorily accounted for the not being able to discover the os uteri or arch of the pubis. I now endeavoured to make the examination in the usual direction, but with more caution; and after repeated attempts I found that the finger passed *readily* into the rectum in every trial.

I *now* learned from Mr. Cooper, that the same circumstance had constantly happened to him during the whole time he had attended the woman, and he had no doubt but the opinion I had formed of the vagina being imperforated was perfectly just; for that some years since he was consulted by this patient for a suppression of urine, when it became necessary to pass the catheter, and he then found the vagina impervious; and on enquiry, he was informed, that "she had had a child, after a very tedious labor, about a year before; was very sore, but got well without mentioning this circumstance to the gentleman who attended her." Mr. Cooper has at various times since mentioned the subject to her mother, who always said, "her daughter was just as *she* was." And so convinced was he of the *impossibility of an impregnation,* that when she spoke to him to attend her, he supposed (without much enquiry or examination) that it would in the end prove to be a case of collected catamenia in the vagina, which might hereafter require surgical assistance; judge then his surprise, when, on being called to this patient, he found her *really in labor.* As he was now informed she had menstruated regularly before this impregnation, he very judiciously supposed that although he could not by an examination find the vaginal aperture, yet from this circumstance there must be one, and that in the natural progress of the labor it might dilate; he therefore waited, finding the strength of the woman kept up, from Tuesday till Friday, when the head pressing down so forcibly and so low, he requested a consultation with Mr. Tice of Ware, and myself.

Finding the circumstances as above described, we were all of opinion, that the parts must in the first instance be minutely examined; that if we could find any opening, (as the woman was said to have menstruated "*the right way*") it should be enlarged; and if we could discover none, that an artificial one should be made, and that then we should wait a reasonable time, to see what effect would be produced by the efforts of Nature.

On inspecting the parts, we found a projection immediately under the meatus urinarius, about the size of half a walnut, and which seemed *a pushing out* of a portion of the vagina, vagina, and directly below this, a slight thin duplicature of the integuments formed by stretching the labia pudendi. Between this ridge and the protrusion above-mentioned, it was natural to expect to find the entrance of the vagina, and as strong a pressure as prudence would allow, was made here for that purpose, but without effect; not the smallest opening could be observed, nor in the *then state* of the parts, could we discern any cicatrix or appearance of adhesion. The perinæum was perfect.

Not being able to find any opening at this part, or by the most accurate examination any communication between the rectum and vagina, we proceeded, according to the opinion we had previously formed, to make an artificial opening. After the contents of the bladder were discharged, a small concave-pointed bistoury was passed on the finger, *within and behind the projection,* through the vagina; and as soon as it was made large enough for the top of the finger, the hairy scalp of the child's head was immediately perceived. The opening was now carefully dilated upwards and downwards, so as to make the orifice about an inch or a little more in length, taking care not to wound the rectum below or the meatus urinarius above. Having now clearly ascertained that it was a natural presentation, that the rectum and meatus urinarius were uninjured, the woman was put to bed and an opiate administered; this was about noon: she was now left to the care of Mr. Cooper, as both myself and Mr. Tice were under the necessity of being absent for some time. I was however, happy in receiving a note from Mr. Cooper the next morning, stating, "that the pains continued very strong till seven o'clock in the evening, before he could perceive any ground gained, when having been out of the room some time, on his return he thought he found a very perceptible difference, and the pains still increasing and the head continuing to advance, about nine she was delivered."

The child was of a full size and still-born.

I visited her by appointment on Sunday, the second day after delivery; we found her without pain or any great tenderness on the abdomen, the skin cool and perspirable, and she had passed her water copiously and without much inconvenience. The pulse was however low and quick; she had two or three rigors, and the tongue had rather a foul appearance. As she had had no motion, the body was directed to be opened by a gentle laxative, and attention paid to prevent adhesion of the divided parts, by a cautious introduction of a piece of candle, or a portion of sheep's gut filled with warm water; it was recommended that for the present she should take a saline mixture with decoction of bark and tinct. opii, and such a portion of wine as might appear to be necessary, and hot flannels sprinkled with brandy were ordered to be occasionally applied over the abdomen.

On Saturday (the ninth day) I was much concerned to hear from my friend Mr. Cooper, "that our patient appeared going on well till Wednesday, when he found her pulse too low and much accelerated, her countenance rather sunk, the lochial discharge, though not copious, yet of a striking fœtor; she made but little complaint, and the belly was not more tender than in common cases. The next day every symptom was aggravated; and notwithstanding all measures were taken to counteract debility, she died on Friday, (the eighth day after delivery)."

As it was impossible to overcome the prejudices of the husband and mother, so as to obtain leave to open the body, we are left to draw our inferences from what is related above; and the first thing observable is, that as the woman had a child *nine years* before, there could *not at that time* be any unnatural obstruction; her time, though stated to be *laborious* and *tedious*, was not more so than commonly happens with first children; she was attended by a woman, and a medical gentleman was called in, but she was delivered before his arrival.

The account that she *was sore afterwards*, makes it reasonable to suppose that ulceration took place after parturition, and that the surfaces of the vagina united by cicatrization, which was the occasion of the present difficulty; and this opinion seems confirmed by Mr. C's account of her when he attended her for a suppression of urine, as, when he passed the catheter, he observed this defect.

Two questions naturally occur here. 1. How did she menstruate, *which it is stated she did*,* and how did the liquor

* The writer is aware how frequently practitioners are deceived, by the description of patients themselves, or their friends, in matters out of the common way; but in the absence of positive facts, these relations are admissible, and particularly if, as in the present case, no practical inference is deduced from them. As a physiological case, the above is certainly defective, as the parts could not be inspected post mortem, but as a practical case it affords some information; and it is principally related with this view. Neither is it certain, that if we could have made the examination after death, that the information we wished for, could have been obtained, as the parts must necessarily have suffered considerable alteration during the progress of labour.

liquor amnii escape as it appeared to have done? 2. How was she impregnated? To the first question I have no satisfactory answer to give, the imperforation of the vagina, as far as we could ascertain by the most accurate examination was complete; neither could we discover by the touch any communication between the rectum and vagina, and the meatus urinarius had a natural appearance. On account of our not being able to obtain leave to inspect the parts after death, we are necessarily left in much uncertainty with respect to this point; and if in respect to this, in how much greater degree must we be in the dark as to the *manner* in which *impregnation took place*? But as I am not willing to wander into the regions of *conjecture*, or to build a fanciful hypothesis on so uncertain a foundation, I shall leave the case in your hands in its present state, vouching only for the accuracy of the facts I have related.

*Bishops Stortford, Herts,* I am, &c.

